# Evaluation of Overall Survival and Disease-Free Survival in Patients Receiving Liver Transplantation for Hepatocellular Carcinoma and Comparison of Living Versus Deceased Donor Liver Transplants: Results of 15 Years of Experience

**DOI:** 10.5152/eurasianjmed.2023.23163

**Published:** 2023-10-01

**Authors:** Ali Avanaz, Haydar Adanir, Abdullah Kisaoglu, Vural Taner Yilmaz, Ezgi Avanaz, Bora Dinc, Ismail Demiryilmaz, Gülsüm Özlem Elpek, Huseyin Kocak, Bulent Aydinli

**Affiliations:** 1 Department of Organ Transplantation, Akdeniz University Faculty of Medicine, Antalya, Turkey; 2 Department of Gastroenterology, Akdeniz University Faculty of Medicine, Antalya, Turkey; 3 Department of Nephrology, Akdeniz University Faculty of Medicine, Antalya, Turkey; 4 Department of Anesthesiology and Reanimation, Akdeniz University Faculty of Medicine, Antalya, Turkey; 5 Department of Pathology, Akdeniz University Faculty of Medicine, Antalya, Turkey

**Keywords:** Disease-free survival, hepatocellular carcinoma, liver transplant, recurrence

## Abstract

**Objective:**

Research comparing patients who received liver transplantation (LT) for hepatocellular carcinoma (HCC) has produced varying outcomes regarding survival and disease-free survival. The objective of this study is to determine the factors that influence the disease-free and overall survivals of those who have undergone LT for HCC and to compare the outcomes of living versus deceased donor liver transplants.

**Materials and Methods:**

We retrospectively analyzed data on patients aged 18 and above who received LT for HCC from 2006 to 2022. Patients with a follow-up period of less than 6 months and who did not meet the University of California San Francisco criteria were excluded. The data from 58 patients were analyzed. We split the patients into living donor liver transplantation (LDLT) (group 1) and deceased donor liver transplantation (DDLT) (group 2).

**Results:**

The mean age was 56 ± 8.1 years. There were 49 males and 9 females. The median of the alpha-fetoprotein (AFP) level and model for end-stage liver disease score was 10.1 ng/mL and 11, respectively. The 1-, 3-, 5-, and 10-year disease-free survival rates were 86%, 76.5%, 76.5%, and 76.5%, respectively. The survival rates for the same periods were 94.8%, 74.9%, 70.6%, and 67.4%. The receiver operating characteristic analysis revealed that AFP > 31.8 ng/mL and a total tumor size >3.85 cm raise the likelihood of HCC recurrence post-LT.

**Conclusion:**

Based on the current literature, the overall survival and disease-free survival rates are influenced by factors such as AFP value, total tumor number, and total tumor diameter. In our study, the AFP value and total tumor size had an impact on the recurrence of HCC, and the survival rates were comparable on LDLT and DDLT.

**Main Points**

- Alpha-fetoprotein level >31.8 ng/mL and total tumor size >3.85 cm have raised the likelihood of hepatocellular carcinoma (HCC) recurrence post-liver transplantation.
- Overall and disease-free survival rates were comparable between living donor liver transplantation and deceased donor liver transplantation.
- Computer-based calculators like “the Metroticket Project” should be implemented to estimate the recurrence rate of HCC.

## Introduction

Liver cancer is the sixth most commonly diagnosed type globally and ranked third in cancer-related deaths. Hepatocellular carcinoma (HCC) and intrahepatic cholangiocarcinoma are the 2 most frequently occurring primary liver cancers, accounting for 75%-85% and 10% of cases, respectively. Several factors increase the risk of developing HCC, including chronic infection with hepatitis B virus or hepatitis C virus, consumption of aflatoxin-contaminated foods, excessive alcohol intake, obesity, type 2 diabetes, and smoking.^1^

The Barcelona Clinic Liver Cancer recommends liver transplantation (LT) for selected stage A and stage B patients in the treatment of HCC.^2^ Liver transplantation involves implanting a graft from a living or deceased donor into a patient. Transplant centers use Milan or expanded criteria to determine which patients with HCC are suitable candidates for LT.^3-6^

Living donor liver transplantation (LDLT) is the more common procedure in Asian countries, while deceased donor liver transplantation (DDLT) is more frequently performed in European and American countries. Research comparing patients who received LDLT and DDLT for HCC has produced varying outcomes regarding survival and disease-free survival.^7-9^

The objective of this study was to determine the key factors that have a significant impact on the prolonged survival and disease-free survival of patients who have undergone LT for HCC. Additionally, the study seeks to compare the long-term survival and disease-free survival rates between patients who underwent LDLT and DDLT.

## Materials and Methods

We retrospectively analyzed data on patients aged 18 and above who received LT for HCC from 2006 to 2022. The ethics committee approval was obtained from the Akdeniz University Institutional Board (26.04.2023/338). Written informed consent was obtained from the living patients. During this period, it was found that 79 patients received LT for HCC. Patients with a follow-up period of less than 6 months and who did not meet the Milan and University of California San Francisco (UCSF) criteria were excluded from the study. We analyzed the data of 58 patients in the study. We divided the patients into LDLT (group 1) and DDLT (group 2) and then compared them. We analyzed various patient factors, including age, gender, transplant etiology, model for end-stage liver disease (MELD) score, largest/total tumor size, tumor number, tumor differentiation (grade), pre-transplant alpha-fetoprotein (AFP) level, pre-transplant bridge therapy, transplant type, recurrence rate, overall survival, disease-free survival, follow-up time, cause of death, and location of recurrence.

### Statistical Analysis

Statistical analysis was performed using the Statistical Package for Social Sciences for Windows version 23.0 (IBM SPSS Corp.; Armonk, NY, USA). Categorical variables were compared by the Pearson’s chi-square or Fisher’s exact tests, and the data are presented as numbers and percentages. Normally distributed continuous data are presented as mean and SD, and non-normal data were presented as the median and interquartile range (IQR). We used the Shapiro–Wilk or Kolmogorov–Smirnov tests to determine the data distribution. We then performed either a Student’s *t*-test or a Mann–Whitney *U*-test, depending on the test assumptions. We used the Kaplan–Meier method to analyze overall and disease-free survival and the log-rank test to compare groups. We also conducted univariate and multivariate Cox regression analyses to identify factors that affect survival. In addition, we employed receiver operating characteristic (ROC) analysis to determine the cutoff point of continuous variables found to be effective in survival analysis. A *P* value of less than .05 was considered to be statistically significant.

## Results

The mean age of the patients was 56 ± 8.1 years. There were 49 (84%) males and 9 (16%) females. The median values for the largest tumor size, total tumor size, and number of tumors were 2.5 (IQR = 2.0-2.8) cm, 2.9 (IQR = 2.0-4.0) cm, and 1 (IQR = 1-1), respectively. The median AFP level and MELD score were 10.1 (IQR = 3.7-70.5) ng/mL and 11 (IQR = 9-17), respectively. The disease-free follow-up time was 58.3 ± 44.5 months, while the overall follow-up time was 62.5 ± 42.8 months. We found that 7 (12%) patients underwent bridge therapy. Hepatocellular carcinoma recurrence was detected in 12 (21%) patients. There were 17 (30%) deaths in this study. The cause of death was HCC recurrence in 11 (19%) patients. Table 1 displays the demographic details of the patients involved in this cohort, while Table 2 shows the locations of recurrence and causes of death.

The number of patients in LDLT (group 1) and DDLT (group 2) was 30 and 28, respectively. We found the mean age was 57 ± 6.6 years in group 1 and 55 ± 9.5 years in group 2 (*P* = .215). There were 5 (17%) females and 25 (83%) males in group 1, while 4 (14%) females and 24 (86%) males in group 2 (*P* = 1.000). In both group 1 and group 2 patients, most cases of cirrhosis were caused by viral hepatitis, with 24 (80%) and 21 (75%) of patients affected, respectively (*P *= .648). The second most common cause was alcohol, affecting 4 (13%) and 3 (11%) of patients, respectively (*P* = 1.000). A small percentage of patients in both groups had cryptogenic cirrhosis, with 2 (7%) patients in each group (*P* = 1.000). No cirrhosis patient was related to other etiologic factors in group 1, whereas 2 (7%) patients had cirrhosis caused by other factors in group 2 (*P* = .229). In group 1, the median AFP value was 16.7 (IQR = 3.4-230.3) ng/mL; in group 2, it was 7.3 (IQR = 4.1-37.5) ng/mL. There was no significant difference between the 2 groups (*P* = .363). The median MELD score was 11 (IQR = 8-16) in group 1, and the mean score was 13 ± 5.3 in group 2 (*P* = .487). According to the tumor characteristics, the number of tumors was 1 (IQR = 1-1.3) and 1 (IQR = 1-1), the largest tumor diameter was 2.8 ± 1.3 cm and 2.8 ± 1.3 cm, and the total tumor diameter was 3.2 ± 1.7 cm and 3 ± 1.6 cm in groups 1 and 2, respectively. Notably, the number of tumors, largest tumor diameter, and total tumor diameter did not exhibit any significant difference between the 2 groups (*P* = .227; *P* = .942; *P* = .363, respectively). Out of the patients in group 1, 9 (30%) had a grade 1, 16 (53%) had a grade 2, and 5 (17%) had a grade 3 tumor. On the other hand, 11 (39%) of group 2 patients had a grade 1, 10 (36%) had a grade 2, and 7 (25%) had a grade 3 tumor. There was no significant difference between groups according to the tumor grade (*P* = .457 for grade 1, *P* = .178 for grade 2, and *P* = .434 for grade 3). The number of patients who underwent bridge therapy was 3 (10%) in group 1 and 4 (14%) in group 2 (*P* = .701). The follow-up time was 46 months (IQR = 24.8-81) in group 1 and 64 months (IQR = 31-101.8) in group 2 (*P* = .173). The disease-free survival was 42 (IQR = 14.5-79.5) months in group 1 and 69.8 ± 51.1 months in group 2, with no statistical difference (*P* = .104). Table 3 shows the data of recipients according to the groups.

**Figure 1.**
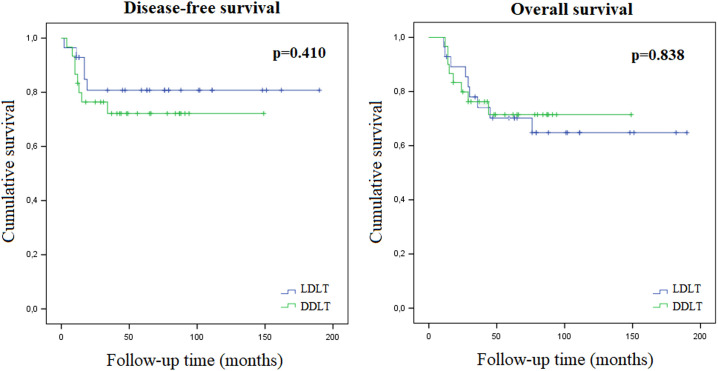
The overall and disease-free survival rates after living donor liver transplantation (LDLT) and deceased donor liver transplantation (DDLT).

**Figure 2.**
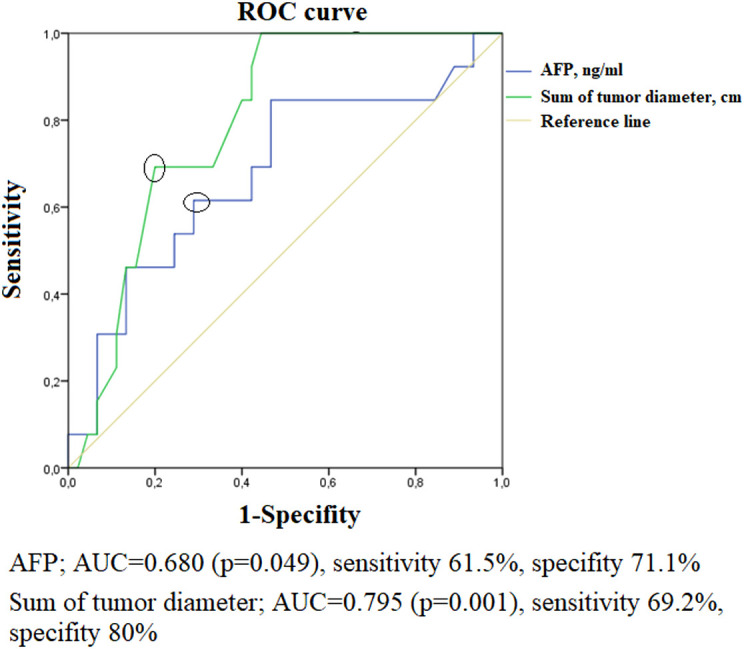
Receiver operating characteristic (ROC) curve analysis of the factors that influenced hepatocellular carcinoma (HCC) recurrence.

In this cohort, the 1-, 3-, 5-, and 10-year disease-free survival rates were 86%, 76.5%, 76.5%, and 76.5%, respectively. The overall survival rates for the same periods were 94.8%, 74.9%, 70.6%, and 67.4%. The 1-, 3-, 5-, and 10-year disease-free survival rates were 83.3%, 72.1%, 72.1%, and 72.1% in group 1 and 92.9%, 80.7%, 80.7%, and 80.7% in group 2, respectively. The survival rates of group 1 and group 2 were analyzed over 1, 3, 5, and 10 years. Group 1 had survival rates of 96.7%, 76.2%, 71.5%, and 71.5%, while Group 2 had survival rates of 92.9%, 74.1%, 70.2%, and 64.8%. The 2 groups had similar survival rates (*P* = .838). Figure 1 displays data on both overall survival and disease-free survival. After examining the factors that affect disease-free survival and overall survival, we found that total tumor size and AFP value negatively impact disease-free survival. We did not find any variable that affects overall survival (Table 4). According to our ROC curve analysis, having a cutoff value for AFP of 31.8 ng/mL and a total tumor size of 3.85 cm raises the likelihood of HCC recurrence post-LT (Figure 2).

## Discussion

Mazzaferro et al^3^ reported that liver transplant patients meeting the Milan criteria had a 4-year survival rate of 75% and a disease-free survival rate of 83%. Yao et al^4^ reported that patients meeting the UCSF criteria had 90% and 75% survival rates after 1 and 5 years, respectively. A study comparing recipients who met Milan and UCSF criteria found no difference in survival rates following LT.^10^ A multicenter study demonstrated that the 10-year survival rates after LDLT in patients within Milan and UCSF criteria were 64.1% and 69.4%, respectively.^11^ In the Asian perspective, overall survival rates ranged from 80% to 85.2% after LT with more expanded selection criteria.^12^ According to the present study, the overall survival rates at 1, 5, and 10 years were consistent with previous studies.

The 5-year recurrence rates were reported at 19% and 6% in patients who underwent LDLT and DDLT for HCC within the UCSF criteria.^7^ The 5-year disease-free survival rate was 79% in LDLT and 75% in DDLT recipients who met Milan criteria and 83% and 71%, respectively, in recipients within the UCSF criteria. It was also reported that the 5-year overall survival rate was 69% in LDLT recipients and 60% in DDLT recipients who met the Milan criteria and 71% and 57%, respectively, in recipients within the UCSF criteria. This study revealed that overall and disease-free survival rates were comparable between LDLT and DDLT.^8^ Additionally, in a recent review that used the data of the United Network for Organ Sharing, it was shown recurrence rate and graft survival rate were similar for HCC after LDLT and DDLT.^13^ In the present study, we found similar results as the previous reports for overall and disease-free survival rates between LDLT and DDLT.

Several risk factors were defined for HCC recurrence after LT. In a study, AFP level >400 ng/mL before LT, microvascular invasion, LDLT, and Edmonson–Steiner grade 3 and 4 tumor differentiation were the risk factors of HCC recurrence.^7^ Banghiu et al^14^ reported that LT beyond UCSF criteria, Edmonson–Steiner grade 3 and 4 tumor differentiation, and microvascular invasion were independent risk factors, while Sandhu et al^9^ reported microvascular invasion was a risk factor for HCC recurrence. Additionally, Fisher et al^15^ reported that AFP level, recipient age, experience at the transplant center, and the era of transplantation were independent risk factors for overall and disease-free survival. Mehta et al^16^ defined the risk estimation of tumor recurrence after transplant (RETREAT) score. They revealed the independent risk factors of recurrence were microvascular invasion, AFP level, and the sum of the largest diameter of viable tumors plus the number of tumors. According to the present study, the AFP value and total tumor size are independent risk factors for HCC recurrence after LT, consistent with previous research. However, we did not identify any risk factors that affect overall survival.

A recent study suggested that AFP levels should be evaluated differently based on the tumor diameter (cm) and number. For tumors with a diameter and number of 7, AFP levels below 200 ng/mL should be considered. For tumors with a diameter and number of 5, levels between 200 and 400 ng/mL are appropriate. Tumors with a diameter and number of 4 should have AFP levels below 1000 ng/mL.^17^ Patients within the Milan criteria with pre-transplant AFP levels above 25.5 ng/mL or detection of an increase in AFP levels above 20.8% on the waiting list were associated with higher recurrence rates.^18^ Furthermore, AFP levels >30 ng/mL and tumor diameter >5 cm were associated with HCC recurrence.^19^ According to the present study, there is a higher risk of HCC recurrence if the AFP level is above 31.8 ng/mL and the tumor size is larger than 3.85 cm.

Although the study’s limitations include being single centered and retrospective, and examining data from a small number of patients, it was strengthened by its ability to provide long-term follow-up results.

Based on the current literature, both LDLT and DDLT procedures have shown similar overall survival and disease-free survival results. The survival rates and disease-free survival rates are influenced by factors such as AFP value, total tumor number, and total tumor diameter. Based on the current literature, both LDLT and DDLT procedures have shown similar overall survival and disease-free survival results. The survival rates and disease-free survival rates are influenced by factors such as AFP value, total tumor number, and total tumor diameter. In our study, we found that both the AFP value and total tumor size had an impact on the recurrence of HCC. Furthermore, we found that the survival rates after LDLT and DDLT were comparable in our experience. When we set the limits of tumor diameter as 4 cm and AFP level to 1000 ng/mL, the estimated 5-year survival is calculated at 60% according to the Metroticket calculator. With the same parameters, the RETREAT score is found to be 5. According to the RETREAT score, 1- and 5-year recurrence risks were reported at 39% and 75% if the score was 5 or higher. However, the RETREAT score covers only the patients within the Milan criteria. Yao et al^4^ reported that the recurrence rate was 11.4% in patients who met the UCSF criteria within a 2-year median follow-up period. When we evaluated the results of the present study and the evidence of the literature, we can conclude that a larger, multicenter study is to be conducted to evaluate more patients and implement a computer-based calculator like “the Metroticket Project” to estimate the recurrence rate of HCC.

## Figures and Tables

**Table 1.** Demographic Information of Liver Transplant Recipients

Age, years	56 ± 8.1
Gender, n (%)	
Female	9 (16)
Male	49 (84)
Model for end-stage liver disease score	11 (9-17)
Alpha-fetoprotein level (ng/mL)	10.1 (3.7-70.5)
Follow-up time, months	62.5 ± 42.8
Disease-free follow-up, months	58.3 ± 44.5
Etiology, n (%)	
Viral	45 (78)
Alcohol	7 (12)
Cryptogenic	4 (7)
Others	2 (3)
Tumor features	
Number	1 (1-1)
Largest diameter, cm	2.5 (2.0-2.8)
Sum of diameter, cm	2.9 (2.0-4.0)
Differentiation	
Grade 1, n (%)	20 (34)
Grade 2, n (%)	26 (45)
Grade 3, n (%)	12 (21)
Bridge therapy, n (%)	
Yes	7 (12)
No	51 (88)

Parametric data are presented with ±SD and non-parametric data are presented with interquartile range.

**Table 2.** The Cause of Death and Location of Recurrence in All Cohort

Cause of Death	n (%)
Lung cancer	2 (3.4)
COVID-19 infection	1 (1.7)
HCV recurrence	1 (1.7)
Myocardial infarction	1 (1.7)
Sepsis	1 (1.7)
HCC recurrence	11 (19)
Location of recurrence	n (%)
Intra-abdominal lymph node	2 (3.4)
Liver	4 (6.9)
Liver + lung	1 (1.7)
Liver + bone	1 (1.7)
Bone	4 (6.9)

COVID-19, coronavirus disease 2019; HCC, hepatocellular carcinoma; HCV, hepatitis C virus.

**Table 3.** Comparison of Variables According to Type of Liver Transplantation

	Living Donor Liver Transplantation (n = 30n = 30)	Deceased Donor Liver Transplantation (n = 28n = 28)	*P*
Age, years	57 ± 6.6	55 ± 9.5	.215
Gender, n (%)			
Female	5 (17)	4 (14)	1.000
Male	25 (83)	24 (86)	
Model for end-stage liver disease score	11 (8-16)	13 ± 5.3	.487
Alpha-fetoprotein level (ng/mL)	16.7 (3.4-230.3)	7.3 (4.1-37.5)	.363
Follow-up time, months	46 (24.8-81)	64 (31-101.8)	.173
Disease-free follow-up, months	42 (14.5-79.5)	69.8 ± 51.1	.104
Etiology, n (%)			
Viral	24 (80)	21 (75)	.648
Alcohol	4 (13)	3 (11)	1.000
Cryptogenic	2 (7)	2 (7)	1.000
Others	-	2 (7)	.229
Tumor features			
Number	1 (1-1.3)	1 (1-1)	.227
Largest diameter, cm	2.8 ±1.3	2.8 ± 1.3	.942
Sum of diameter, cm	3.2 ±1.7	3 ± 1.6	.557
Differentiation			
Grade 1, n (%)	9 (30)	11 (39)	.457
Grade 2, n (%)	16 (53)	10 (36)	.178
Grade 3, n (%)	5 (17)	7 (25)	.434
Bridge therapy, n (%)			
Yes	3 (10)	4 (14)	.701
No	27 (90)	24 (86)	

Parametric data are presented with ±SD and non-parametric data are presented with interquartile range.

**Table 4.** The Associated Factors with Disease-free Survival

Univariate Analysis			
Variable	HR	95% CI	*P*
Largest tumor diameter, cm	1.7	1.1-2.5	**.01**
Sum of tumor diameter, cm	1.4	1.1-1.9	**.009**
**Multivariate Analysis**			
**Variable**	**HR**	**%95 CI**	***P***
AFP level (ng/mL)	1.0	1.0-1.0	**.043**
Sum of tumor diameter, cm	1.5	1.1-2.0	**.006**
